# Updating the Genome of the Elite Rice Variety Kongyu131 to Expand Its Ecological Adaptation Region

**DOI:** 10.3389/fpls.2019.00288

**Published:** 2019-03-13

**Authors:** Rongsheng Wang, Guoqiang Jiang, Xiaomin Feng, Jianzong Nan, Xiaohui Zhang, Qingbo Yuan, Shaoyang Lin

**Affiliations:** ^1^University of Chinese Academy of Sciences, Beijing, China; ^2^State Key Laboratory of Plant Genomics, Center for Genome Biology, Institute of Genetics and Developmental Biology, Chinese Academy of Sciences, Beijing, China

**Keywords:** elite rice variety, heading date, SNP marker, QTL, gene module

## Abstract

As an elite rice variety cultivated in the third accumulative temperature belt in Heilongjiang province, China, Kongyu131 has many excellent traits, such as high quality, high stability, early maturation and cold resistance. However, as with other crop varieties, Kongyu131 has regional restrictions, exhibiting decreased yields when grown at low latitudes. To address these problems, two populations were constructed from cross between *japonica* and *indica* varieties. QTL analyses were performed with these two populations to detect regional adaptation related quantitative trait locus. Results in a BC_1_F_6_ backcross inbred line population with 168 lines derived from cross between Kongyu131 and GKMP showed a large pleiotropic QTL near 9 Mb on chromosome 7, which significantly delayed the HD of Kongyu131 and increased the plant height (PH), length of main panicle (LMP), number of primary branches (NPB) and grain number of main panicles (GNP). We also found a similar QTL in the population BC_3_F_2_ derived from Kongyu131 and GKLPL. Based on the QTL, we developed a gene module named mRA7 with 5 single-nucleotide polymorphism (SNP) markers around the QTL. Through a foreground and background selection based on 197 SNP markers evenly distributed over the 12 chromosomes, we obtained a new plant (a single point substitution line, SPSL) with a new Kongyu131 genome, carrying only a small chromosomal fragment less than 800 kb from GKLPL. The background recovery ratio of the SPSL was 99.8%. Compared with Kongyu131, the SPSL exhibited a significant HD delay of approximately 31 days and increased PH, LMP and GNP values when planted in Heilongjiang province. When cultivated in Guangdong province, HD of SPSL showed only 16 days delay, and less increase in PH, LMP and GNP than in Heilongjiang province. Phenotypic evaluation showed that the SPSL could be moved to south by more than 3 latitude units and cultivated in low-latitude regions. This study exemplifies the feasibility of expanding the regions of cultivation of elite rice varieties via similar methods.

## Introduction

Successful breeding of an elite variety requires a tremendous amount of time and resources. Spreading of a main cultivar to other cultivation regions could have great significance in saving resources. However, as with other crop varieties, rice varieties exhibit specific regional adaptability, which is mainly observed as abnormal heading dates (HDs) and plant architecture when cultivated elsewhere. As a consequence, a specific rice variety can be planted in only a limited cultivation region with specific photoperiod and temperature regime across the growth cycle. Once outside its adaptive environment, a rice variety may grow excessively without heading or prematurely with reduced yield and quality. Regional adaptability has become the main problem restricting the introduction of elite rice varieties to other cultivation regions.

The main determinant of the regional adaptation of rice to different environments is the transition period from vegetative growth to reproductive growth, also known as the heading date (Hayama et al., 2003; Wang et al., 2011). Furthermore, plant height (PH) and length of the main panicle (LMP) are key indicators of adaptation and can be used to decide whether a certain variety can be cultivated elsewhere (Xing and Zhang, 2010). As a short-day plant, rice always exhibits early flowering at low latitudes but delayed flowering at high latitudes. A series of heading date-related quantitative trait loci (QTLs) and genes have been identified, such as *Hd1* (Yano, 2000), *Hd3a* (Kojima et al., 2002; Tamaki et al., 2007), *Ehd1* (Doi et al., 2004), *Ghd7*/*Hd4* (Yano et al., 1997; Xue et al., 2008), *Hd6* (Takahashi et al., 2001), and *Hd16* (Hori et al., 2013). However, there have been only a limited number of reports regarding the improvement of the regional adaptability of rice to environments at high latitudes by manipulation of HD-related QTLs or genes (Yu et al., 2002; Nagata et al., 2015). Although rice varieties in northeast China are famous for their high quality, few of these varieties have been extended to the south. Indeed, all the genetic modifications necessary to improve the adaptation capacity are limited by improvement accuracy. In addition neither traditional breeding nor marker-assisted selection (MAS)-based breeding technology can adjust the HD of a rice variety without changing other excellent traits (Kwon et al., 2002; Zhou et al., 2003). A precise genetic technique is urgently needed to address such problems.

With the rapid development of plant genomics, as whole-genome resequencing and molecular marker technology, we now have an opportunity to address these issues. First, an increasing number of excellent genetic resources regarding natural variations associated with HDs have been identified and compiled for rice breeding (Fan et al., 2006; Zhang et al., 2012; Tang et al., 2013). The dissection of complex HD networks has provided new perspectives for the molecular design of new cultivars with modified photoperiod adaptability that can effectively grow in different cultivation regions (Yano and Sasaki, 1997; Xing and Zhang, 2010; Matsubara et al., 2014). Furthermore, with the development and application of next-generation sequencing and interpretation of the large amounts of data generated (Duitama et al., 2015; Wang et al., 2018), single-nucleotides polymorphisms (SNPs) (Chen et al., 2014; Yu et al., 2014) coupled with high-resolution melting (HRM) analysis for genotyping can improve the ease and effectiveness of the detection of molecular markers. In 2003, “breeding by design” was first proposed as a new breeding strategy (Peleman and van der Voort, 2003). Following with this concept, many new attempts were carried out in recent years (Wang et al., 2011; Zeng et al., 2017). But for the precise and efficient breeding, a gene module, that is a foreign short chromosome segment regulating relevant agronomic trait(s), should be defined together with corresponding SNP markers. With modules developed and the technical foundation mentioned above, the improvement of new variety and aggregation of multi module would be feasible.

Kongyu131 is an elite rice variety of the third accumulated temperature belt in Heilongjiang province, China. This variety has many advantageous traits, such as high quality, high stability, early maturation and cold resistance, which have led to increasing its annual cultivation area since 1990. The cultivation area peaked in 2004, occupying more than half of the total rice cultivated area in Heilongjiang province, and its cumulative extension area has accounted for more than 9.5 million hectares to date (China Rice Data Center, 2018). Kongyu131 exhibits significantly premature development with dramatic height and yield reduction when cultivated in low-latitude regions such as Beijing. In general, increasing adaptation capacity of elite rice varieties by genetic modification of their genome could have a relevant impact for the breeding sector.

In this study, we carried out QTL analysis in two backcross populations and identified a pleiotropic and stable QTL to repair this “bug” in Kongyu131 when grown at low latitudes. After genomic background subtraction, a single point substitution line (SPSL) containing a target chromosome segment was bred. We tested the SPSL in different rice fields and proved that it can be moved to southern regions.

## Materials and Methods

### Plant Materials and Field Management

The recurrent parent used in this study was Kongyu131, an elite *japonica* rice variety that is widely cultivated in the third accumulated temperature belt in Heilongjiang province, China. Kongyu131 is an early maturation variety, with seeding to heading occurring over 98 days, and requires at least 2320°C as an active accumulated temperature. Kongyu131 has an approximate PH of 80 cm, LMP of 16 cm, and GNP of 109. The donor parent GKMP, is an *indica* cultivar bred from Modan and Koshihikari, which is the main cultivar in Japan. GKMP is not able to head in Heilongjiang province. When cultivated in Beijing, GKMP showed early maturation that required 104 days from seeding to heading, PH of 90 cm, LMP of 20 cm and GNP of 156. The other donor parent GKLPL is also an *indica* cultivar bred from GKMP but with a different plant type. When cultivated in Heilongjiang province, GKLPL showed late maturation that required 122 days from seeding to heading. This variety had an approximate PH of 96 cm, LMP of 27 cm, and GNP of 286.

All phenotypic experiments were carried out as follow. Presoaking of seeds was conducted in an 8^∗^12-well plate with a small hole at the bottom. We used 96-well plate with 96 plants as a plot. After sowing, the plates were immersed in water at a constant temperature of 37°C and germinated for approximately 48 h. The seeds were transplanted to nutrient soil to obtain seedlings in bed boxes. Seedlings at the trefoil stage were transplanted to the paddy field in the same arrangement as that in the 96-well plate with one plant per hole, and the plants were spaced with 20 cm within each row and 30 cm between adjacent rows. The area per plot was 5.76 m^2^ (2.4 m × 2.4 m). The other conditions were the same as those used for local paddy field management.

### Population Construction and Selection of the SPSL

Kongyu131 was crossed with GKMP, the F_1_-generation was backcrossed with Kongyu131 to produce the BC_1_F_1_-MP population. One seed of each BC_1_F_1_-MP line was picked to obtain its inbred seed with a random selection in every generation as according to SSD procedure. Finally, we obtained the backcross inbred line population BC_1_F_6_-MP with GKMP as the donor for a total of 168 lines BC_1_F_6_-MP. This population was used for QTL analysis.

**FIGURE 1:**
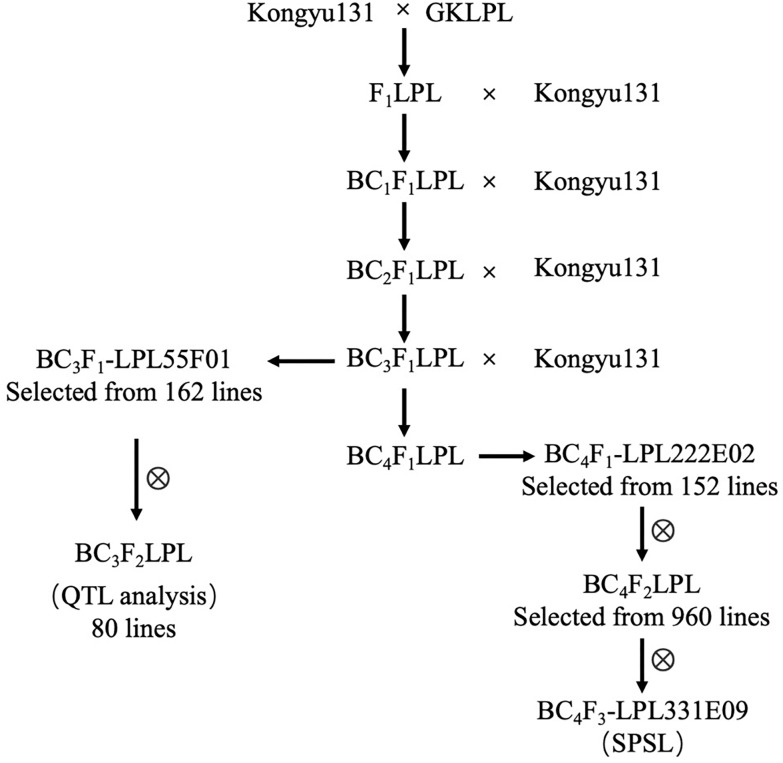
Construction of the population used for modular introduction.

Kongyu131 was crossed with GKLPL, and then, the F_1_-generation was backcrossed with Kongyu131 consecutively to produce the BC_3_F_1_-LPL population for a total of 162 lines (Figure 1). From this population, we selected a line named BC_3_F_1_-LPL55F01. 80 progeny plants generated from the selfing of BC_3_F_1_-LPL55F01 were transplanted to the paddy field at the seedling stage and phenotyped in Jiamusi, Heilongjiang province for HD, LMP, and PH and scoring data used for QTL analysis. Simultaneously, one plant from each line of the BC_3_F_1_-LPL population was backcrossed with Kongyu131 producing the BC_4_F_1_-LPL population of 152 lines. This BC_4_F_1_-LPL population was cultivated and genotyped with SNP markers for genotypic selection. Then, based on the genotypic data we selected a plant named BC_4_F_1_-LPL222E02, in which a crossover occurred within SNP1 and SNP2 and contained the target QTL of the donor. The nine hundred and sixty progenies obtained by selfing of the BC_4_F_1_-LPL222E02 plant were genotyped for markers in the region comprising SNP2 – SNP5. Based on this molecular analysis, plants heterozygous at these markers were selected for genotypic selection in Hannan province. Finally, an SPSL plant named BC_4_F_3_-LPL331E09 was obtained. In this plant, a crossover occurred within SNP4 and SNP5, but the homozygous segment between SNP2 to SNP4 were from the donor GKLPL.

### Trait Investigation and Data Analysis

The populations used for QTL analysis were planted in Beijing (ZGQ, 39.5°N, 116.5°E) and Jiamusi, Heilongjiang province (LQ, 46.5°N, 130.5°E), separately. Phenotypical validation was carried out in LQ, Changchun, Jilin province (JNQ, 43.5°N, 125.5°E), ZGQ and Guangzhou, Guangdong province (GZQ, 20.5°N, 110.5°E). Different sowing date experiment was carried out in ZGQ. In all field environments, the selfing descendants of the SPSL BC_4_F_3_-LPL331E09 and its parents were planted in a randomized block design with three replications. Eight plants in each plot were randomly selected for phenotypic evaluation. The heading date of each plant was recorded when its panicle heading over one third of the panicle length. All agronomic traits were measured three times and followed the standard evaluation system (International Rice Research Institute [IRRI], 2013), including PH, HD, LMP, NPB, GNP, ETP, GN and 1000-grain weight (TGW). Days to heading (DH) was counted from sowing date to heading date. Then, average correlation coefficient and Student’s *t*-tests were calculated in MS Excel. Arithmetic average with standard deviations were calculated as the phenotype of the SPSL and its parents with 8 plants per replication. Broad-sense heritability (*h*_B_^2^) was calculated with the formula *h*_B_^2^ = V_G_/V_P_.

### Molecular Assay and Analysis

#### DNA Isolation and HRM

Genomic DNA was extracted following an improved rapid high-throughput DNA isolation method (Stein et al., 2001; Paris and Carter, 2012). One hundred and sixty microliters of precooled Tris–HCl (0.1 M, pH 7.5) and 40 μL of NaOH (0.4 M) were added to a 96-deep-well plate. A piece of fresh leaf was added to the solution buffer with a small steel ball; the plate was covered with a silica gel lid and placed in a vibrator for 3 min; and then, the supernatant was extracted for subsequent polymerase chain reaction (PCR) and high-resolution melting (HRM) analysis after centrifugation.

Polymerase chain reactions were performed in a normal 96-well plate. The reaction system contained 1 μL of 10 × Buffer, 0.4 μL of 2.5 mM dNTPs, 0.1 μL of Taq DNA polymerase, 0.2 μL of 20 × qPCR DNA-binding dye, 10 μM forward primer, 10 μM reverse primer, and 3 μL of genomic DNA template, and ddH_2_O was added to 10 μL. PCR and HRM analysis were implemented in a qPCR instrument continuously. The PCR procedure consisted of predenaturation at 94°C for 2 min, followed by 40 cycles of 94°C for 10 s and 60°C for 30 s. The HRM procedure consisted of predenaturation at 94°C for 10 s, 60°C for 10 s, and heating to 94°C, with 10 instances of fluorescence detection for every degree of temperature increase.

#### Genotyping for the Development of the Genetic Map

For the QTL analysis on the population BC_1_F_6_-MP, a total of 168 SNP markers evenly distributed on 12 chromosomes were used in 168 individuals. According to the genotype of 195 SNP markers evenly distributed on 12 chromosomes, we screened the BC_3_F_1_-LPL population with 162 individuals. An individual named BC_3_F_1_-LPL55F01 was used to derive BC_3_F_2_-LPL population with its self-bred seeds. Only 37 SNP markers were heterozygous in this population. So, we conducted the QTL analysis with these markers in 80 individuals. Genetic distance were calculated in MapMaker/Exp 3.0b and QTL analyses were conducted by MapMaker/QTL 1.1b (Lander et al., 1987).

#### Genotyping for Individual Selection

Individual selections were performed with SNP1 to SNP5, and SNP3 was detected to make sure about the introduction of the gene module. To excluding segments from the donor, the SNP marker located on the other chromosomes were detected if necessary.

#### Resequencing and Design of the Primers

The parents, namely, Kongyu131, GKMP and GKLPL, were resequenced by a HiSeq2000 sequencer. The *Ghd7* DNA sequence was downloaded from GenBank ^1^, and the reference sequence used for genomic assemble and gene alignments was downloaded from Oryzabase^2^ (Nipponbare Build v.4). Sequence search and alignment were conducted using the Localblast system developed by NCBI^3^.

**FIGURE 2:**
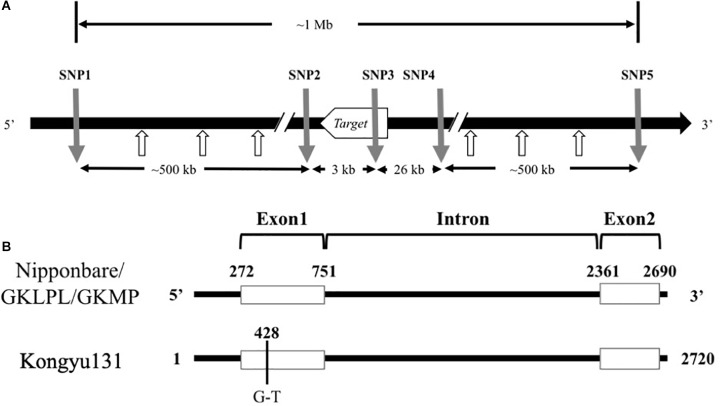
**(A)** Alignment of Ghd7 sequences from Nipponbare, GKLPL and Kongyu131. The number indicates the position of the Ghd7sequence. The black line indicates the intron region, and the white box indicates the exon region. **(B)** The relative positions of the SNP markers used for introduction of the mRA7 module. The gray arrows indicate the positions of five SNP markers used for introduction of the module, and the white arrows indicate the SNP marker used for detection of module length.

Based on the sequence alignment between the two parents, some effective SNP positions were selected for SNP marker design. Genome sequences around SNP position were uploaded as supplementary data (SupplementaryData.xlsx). Primers of 18–25 nts were selected around the SNP positions and to give a PCR product with a 40–100 bp length. The SNP markers for the mRA7 module, named SNP1 to SNP5, were located upstream (SNP1 and SNP2), within (SNP3), and downstream (SNP4 and SNP5) of this gene (Supplementary Table S1). The distance from SNP1 to SNP5 was approximately 1 Mb (Figure 2A).

#### Development of the Genetic Maps

The genetic maps were performed by MapChart 2.32 (Voorrips, 2002). 168 SNP were used for the BC_1_F_6_-MP population. 35 SNP were used for the BC_3_F_2_-LPL population. Each of the only one SNP marker on the 3th and the 4th chromosome was omitted.

## Results

### QTL Analysis of a BIL Population BC_1_F_6_-MP

For QTLs controlling HD useful for improving Kongyu131’s adaptation, we obtained a backcross inbred line (BIL) population BC_1_F_6_-MP (168 individuals) using Kongyu131 as the recurrent parent and GKMP as the donor. After planting the population in a paddy field in ZGQ, we surveyed the phenotypes of these individuals by accessing these lines for DH, PH, effective tillers per plant (ETP), LMP, number of primary branches (NPB) and grain number of main panicles (GNP) after maturity. Frequency distributions of different traits showed nearly normal distributions (Supplementary Figure S1). Heritability ranged from 86.28 to 98.77%, which mean the existence of significant effect of genotypic variation on the phenotypic variation (Table 1). A total of 168 SNP markers were used to carry out the QTL analysis. The total length of the linkage map was 4326.8 cM with an average interval of 25.75 cM (Supplementary Figure S2). We found a pleiotropic QTL near 9 Mb on the 7th chromosome. PH, HD, LMP, NPB and GNP were affected by this QTL. The phenotypic explained variance ranged between 40.90 and 59.60% while LOD score ranged from 19.18 to 33.09 indicating that a major effect QTL with a high confidence level was detected (Supplementary Figure S3). In this QTL region, the gene Ghd7 has been identified. The segment derived from the donor GKMP could significantly delay the HD of Kongyu131 and increase the PH, LMP, NPB and GNP (Table 2).

**Table 1:** Statistics of the analyzed traits in the two populations used for QTL analysis.

Trait	Population	Mean	Min	Max	CV (%)	*h*_B_^2^ (%)^a^
PH	BC_1_F_6_-MP	55.90	33.00	112.00	23.41	98.77
DH	BC_1_F_6_-MP	71.15	52.00	112.00	19.74	97.48
LMP	BC_1_F_6_-MP	16.41	8.80	24.00	20.20	86.28
NPB	BC_1_F_6_-MP	9.17	3.00	18.00	39.19	87.09
GNP	BC_1_F_6_-MP	94.21	22.00	229.00	48.19	94.79
LMP	BC_3_F_2_-LPL	16.51	13.00	20.00	9.14	62.32
PH	BC_3_F_2_-LPL	104.71	82.00	120.00	8.72	97.58
DH	BC_3_F_2_-LPL	125.99	108.00	141.00	6.81	96.83

*DH, Days to heading; PH, plant height; LMP, length of main panicle; NPB, number of primary branches; GNP, grain number of main panicles. ^a^h_B_^2^ (%), broad-sense heritability.*

**Table 2:** Results of the QTL analysis for analyzed traits in BC_1_F_6_-MP population.

Trait	Chr.	Nearest marker	Additive effect	Dominant effect	PVE (%)	LOD score
PH	7	MP07-09	8.57	3.42	40.90	19.18
DH	7	MP07-09	11.13	0.96	59.60	33.09
LMP	7	MP07-09	2.22	1.19	42.80	20.39
NPB	7	MP07-09	2.75	-0.05	55.50	29.54
GNP	7	MP07-09	33.48	32.64	51.50	25.73

*DH, Days to heading; PH, plant height; LMP, length of main panicle; NPB, number of primary branches; GNP, grain number of main panicles; PVE, total phenotypic variance explained; LOD score, likelihood of odds. Additive effects, dominant effects and PVE were calculated by MapMarker/QTL.*

Correlation analysis of traits in this population showed that DH, PH, LMP, NPB, and GNP have a significant relationship between each two of them, all their significant coefficient values were higher than 0.7. ETP showed little relationship with other traits and all their significant coefficient values were lower than 0.25 (Table 3). These suggested the identical conclusion we got from the QTL analysis.

**Table 3:** Correlation of different traits on QTL analysis population BC_1_F_6_-MP.

	PH	DH	ETP	LMP	NPB
DH	0.8513	1			
ETP	0.1934	0.1938	1		
LMP	0.7751	0.7293	0.2219	1	
NPB	0.7951	0.8672	0.2173	0.7776	1
GNP	0.8271	0.8122	0.1640	0.8590	0.8730

*DH, Days to heading; PH, plant height; LMP, length of main panicle; NPB, number of primary branches; GNP, grain number per panicle; ETP, effective tillers per plant.*

### QTL Analysis of an BC_3_F_2_-LPL Population

**FIGURE 3:**
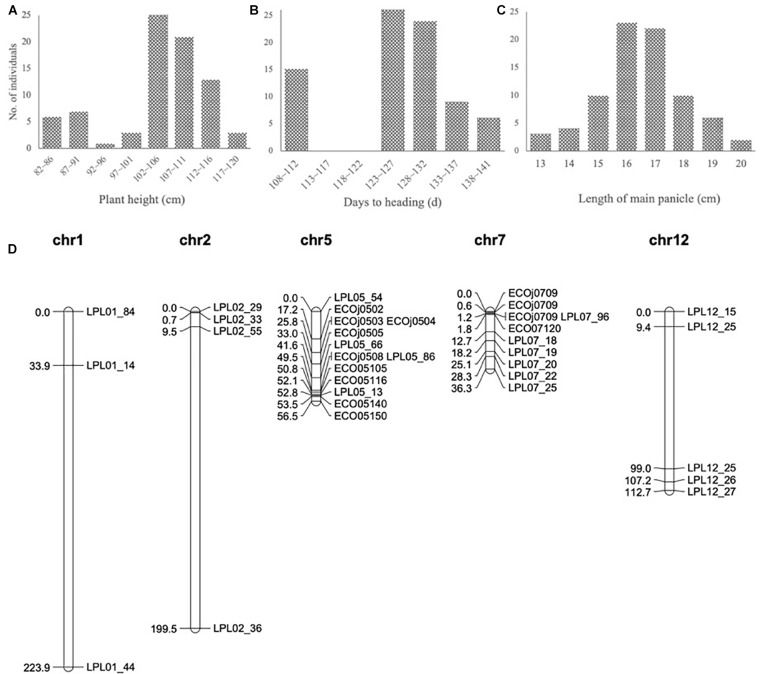
**(A–C)** Frequency distribution in the QTL population BC_3_F_2_-LPL. The vertical axis indicates the number of plants; the horizontal axes indicates the **(A)** intervals of different plant heights, **(B)** intervals of different days to heading and **(C)** intervals of different length of main panicle. **(D)** Genetic map of QTL population BC_3_F_2_-LPL.

To develop the fine mapping and introgression line, a QTL mapping population BC_3_F_2_-LPL was constructed between Kongyu131 and GKLPL. The parent of this population named BC_3_F_1_-LPL55F01 was selected for the heterozygous segment on the short arm of the 7th chromosome (Supplementary Figure S4A). 80 descendants of this line were phenotyped in a paddy field in LQ, in the normal season in 2015. According to the phenotypic statistics of the population, the HD and PH exhibited obvious segregation based on statistics and analysis (Figure 3A,B), but the LMP showed a normal distribution (Figure 3C). The heritability of LMP, PH, and DH ranged from 62.32 to 97.58% (Table 1). It is inferred that the introduced segment from the donor GKLPL could lead to the delay of HD and the increase of plant height, similar to the QTL from GKMP. Therefore, we conducted QTL analysis using 37 SNP markers which showed to be heterozygous in the BC_3_F_1_-LPL55F01 plant (Figure 3D). Finally, a major QTL controlling DH, PH and LMP was located at the 9 Mb position on the short arm of 7th chromosome, as reported for the BIL population BC_1_F_6_-MP (Supplementary Figures S4B–D). This locus explained 86.2% of the phenotypic variance of DH and 71.4% of PH (Table 4). This suggested that the introgression of this locus can lead to delayed HD, and increased PH and LMP in Kongyu131. This QTL located in the same position of a previously reported QTL affecting DH, PH and LMP and cloned as *Ghd7* gene (Yano et al., 1997; Xue et al., 2008).

**Table 4:** Results of the QTL analysis for analyzed traits in BC_3_F_2_-LPL population.

Trait	Marker	Additive	Dominant	PVE%	LOD
HD	GKLPL07-09	11.760	4.867	86.20	34.369
LMP	GKLPL07-09	1.186	0.675	29.80	6.155
PH	GKLPL07-09	10.740	7.589	71.40	21.706

*HD, heading date; PH, plant height; LMP, length of main panicle; PVE, total phenotypic variance explained; LOD score, likelihood of odds. Additive effects, dominant effects and PVE were calculated by MapMarker/QTL.*

Correlation analysis of these three traits used in the QTL analysis showed that LMP has a relationship with PH and DH. PH and DH have a significant relationship with the significant coefficient value 0.8 (Supplementary Table S2).

### Alignment of Gene Sequences Between Parents

To reveal whether the phenotypic changes in these QTL populations were caused by the difference of inferred gene sequence, we firstly conducted genome resequencing in the parents Kongyu131, GKLPL and GKMP. BLAST analysis of the *Ghd7* gene sequences of these varieties, using the whole-genome sequence of Nipponbare as a reference, showed that this gene was located near 9 Mb on chromosome 7, similar to the location of this gene in Nipponbare, thus being a reliable candidate gene for the QTL. Alignment of the *Ghd7* gene sequences of these parents exhibited a single base mutation, from G to T, in the first exon of Kongyu131 *Ghd7* at position 428 (Figure 2B). The mutation led to a change from GAG, encoding glutamic acid (Glu), to TAG, which is a stop codon. This mutation may lead to loss of function of this gene. The gene sequences in GKLPL and GKMP were identical.

To ensure precise introduction of the target gene, we designed five SNP markers, namely, SNP1 to SNP5, around this gene according to the sequence alignment information. SNP3 was located in the gene and designed based on the mutant base site. SNP1 and SNP2 were located upstream of the gene, while SNP4 and SNP5 were located downstream (Figure 2A).

### Introduction of the Donor Chromosome Segment (Defined as the Gene Module) and Size Estimation

To reduce the extension of the introgressed region, upon a further backcross a BC_4_F_1_-LPL population of 152 individuals was generated and genotyped with 197 SNP markers evenly distributed on 12 chromosomes. An individual named BC_4_F_1_-LPL222E02, with a donor chromosome segment on 7th chromosome and with the least segments on the other chromosomes was selected. In addition to the introduced target segment, it carried also some segments on chromosomes 1, 3, and 7, and the total length of the introduced segments was approximately 28 Mb. Detection of SNPs from SNP1 to SNP5 showed that SNP1 originated from Kongyu131, indicating that a crossover occurred between SNP1 and SNP2. The background recovery ratio was approximately 97.5% (Supplementary Figure S5A).

To further eliminate the linkage drag downstream of the target gene module, nine hundred and sixty progenies of the self-bred BC_4_F_1_-LPL222F02 were sowed and screened at the seedling stage with four SNP markers (SNP2 to SNP5) to select an individual in which recombination occurred between SNP4 and SNP5 while ensuring that the SNP3 position originated from the donor. Then, these selected individuals were transplanted to a normal paddy field to obtain self-bred seeds. The same procedure was performed in BC_4_F_2_-LPL and BC_4_F_3_-LPL population for excluding the segments from the donor, except the target gene module. Finally, we obtained an SPSL named BC_4_F_3_-LPL331E09. The segment between SNP2 and SNP4 was homozygous and originated from the donor GKLPL. This gene module was named mRA7. All the other chromosomes were recovered from Kongyu131 with a 99.8% background recovery ratio (Supplementary Figure S5B).

To accurately determinate the length of the introduced sequence, we designed 3 SNP markers between SNP1 and SNP2 as well as between SNP4 and SNP5 separately, with an average marker to marker distance of approximately 120 kb (Figure 2A). We found that all the SNP markers between SNP1 and SNP4-2 exhibited homozygous genotypes from GKLPL, indicating that the segment introduced was shorter than 800 kb.

### Phenotype Comparison Between the SPSL and Kongyu131 in Different Environments

**FIGURE 4:**
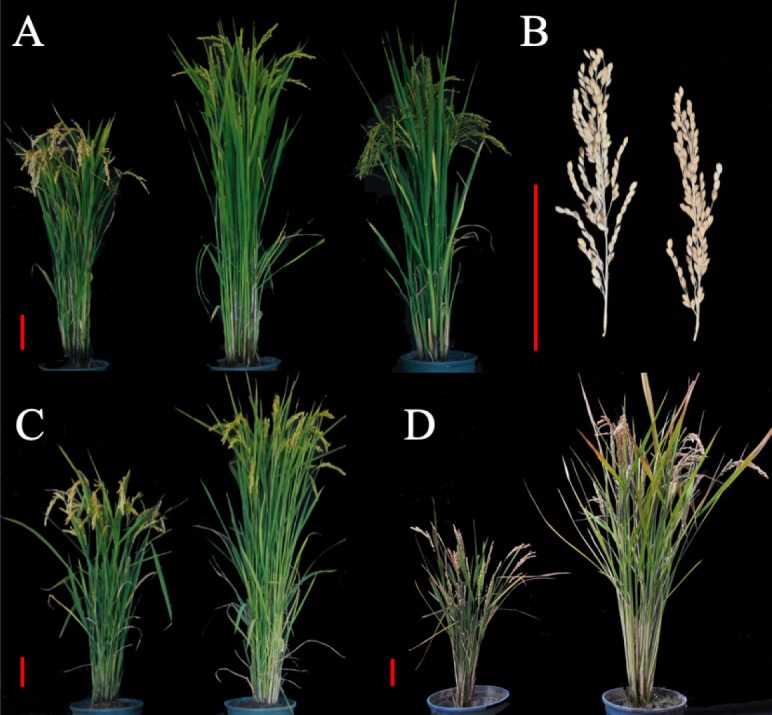
Phenotype comparison between SPSL and Kongyu131 in different environments. **(A)** Kongyu131 (left), SPSL (middle) and GKLPL (right) planted in LQ. The photo was taken on September 3, 2017, and the SPSL exhibited a higher plant height than Kongyu131 and a late heading date. **(B)** Comparison of the length of the main panicle between the SPSL (left) and Kongyu131 (right). The SPSL exhibited a significantly longer main panicle than Kongyu131. **(C)** Kongyu131 (left) and the SPSL (right) planted in JNQ. The photo was taken on September 17, 2017, and the SPSL seemed to have undergone maturations. **(D)** Kongyu131 (left) and the SPSL (right) planted in ZGQ. The photo was taken on September 7, 2017. The scale bar represents 10 cm.

LQ was not suitable for cultivation of the SPSL due to the prolonged growth period, so SPSL with its parents were compared simultaneously in four environments – LQ, JNQ, ZGQ and GZQ – to analyze phenotypic variance among these lines. In LQ, the HD was delayed by approximately 31 days; the PH increased by 20 cm; the LMP increased by 1 cm; the NPB increased by 6; and the GNP increased by 31 grains. This indicated that introduction of the mRA7 module indeed delayed the HD and increased the PH and LMP (Figure 4A,B) with a positive effect on GNP. Notably, the SPSL exhibited nearly complete maturation when planted in JNQ and was also suitable for the cultivation season in ZGQ (Figure 4A,C,D). However, a decrease in PH was observed in GZQ due to the early HD, which was nearly 27 cm lower than the value of SPSLs in JNQ and LQ. The DH of SPSL was approximately 84 days when planted in ZGQ, 15 days longer than Kongyu131.

Phenotypic comparison suggested that the DH, PH, LMP, NPB and GNP of the SPSL exhibited significant differences compared with Kongyu131, while for ETP significant differences were found only in GZQ. With decreasing latitudes the HD, PH, NPB, GNP and ETP of the SPSLs all exhibited reduction trend (Table 5).

**Table 5:** Phenotype statistics for the two varieties and the SPSL planted in different areas.

Environment	Trait	Kongyu131	SPSL	GKLPL
LQ	DH	98.46 ± 1.91	128.58 ± 1.50^∗∗^	122.50 ± 1.41
LQ	PH	80.51 ± 1.57	100.15 ± 3.03^∗∗^	96.39 ± 2.53
LQ	LMP	16.00 ± 0.89	17.21 ± 0.59^∗∗^	27.30 ± 1.80
LQ	NPB	10.25 ± 1.19	16.29 ± 0.86^∗∗^	16.63 ± 1.21
LQ	GNP	105.13 ± 10.33	137.79 ± 16.99^∗∗^	285.46 ± 42.18
LQ	ETP	29.58 ± 3.24	29.25 ± 2.51	12.25 ± 2.54
LQ	GN	2280.96 ± 333.51	2900.17 ± 447.22^∗∗^	–
LQ	TGW	27.42 ± 0.81	23.50 ± 0.58^∗∗^	–
JNQ	DH	99.58 ± 1.89	125.29 ± 1.71^∗∗^	120.54 ± 2.04
JNQ	PH	71.14 ± 2.57	101.50 ± 2.29^∗∗^	102.13 ± 1.63
JNQ	LMP	13.42 ± 1.08	16.15 ± 1.07^∗∗^	34.18 ± 1.23
JNQ	NPB	9.25 ± 0.94	15.71 ± 1.00^∗∗^	15.71 ± 1.08
JNQ	GNP	94.29 ± 20.88	135.33 ± 16.97^∗∗^	299.08 ± 29.01
JNQ	ETP	28.29 ± 2.77	27.79 ± 3.99	12.29 ± 1.73
ZGQ	DH	69.29 ± 1.49	83.79 ± 1.74^∗∗^	92.38 ± 1.28
ZGQ	PH	63.86 ± 2.31	81.50 ± 4.85^∗∗^	99.75 ± 2.54
ZGQ	LMP	13.67 ± 0.62	17.48 ± 1.41^∗∗^	34.70 ± 2.22
ZGQ	NPB	7.17 ± 0.96	12.67 ± 2.51^∗∗^	15.75 ± 1.33
ZGQ	GNP	71.38 ± 10.13	131.17 ± 22.08^∗∗^	359.96 ± 51.70
ZGQ	ETP	26.71 ± 2.56	27.00 ± 4.28	10.08 ± 1.93
ZGQ	GN	1379.92 ± 249.18	2200.42 ± 571.99^∗∗^	3145.79 ± 546.11
ZGQ	TGW	23.95 ± 0.72	23.45 ± 1.39	27.26 ± 1.49
GZQ	DH	62.13 ± 2.25	78.21 ± 2.28^∗∗^	72.38 ± 1.86
GZQ	PH	64.63 ± 2.32	72.88 ± 3.91^∗∗^	94.55 ± 2.61
GZQ	LMP	13.66 ± 1.01	15.28 ± 1.11^∗∗^	35.48 ± 2.11
GZQ	NPB	7.88 ± 0.85	11.54 ± 1.64^∗∗^	13.38 ± 1.21
GZQ	GNP	69.33 ± 7.82	91.13 ± 10.30^∗∗^	225.83 ± 51.90
GZQ	ETP	17.83 ± 1.93	13.63 ± 2.22^∗∗^	6.00 ± 1.29

*DH, Days to heading; PH, plant height; LMP, length of main panicle; NPB, number of primary branches; GNP, grain number of main panicles; ETP, effective tiller per plant; GN, grain number per plant; TGW, 1000-grain weight. Data presented as Means ± SD. n = 8 ^∗^ 3 = 24. ^∗∗^: represents significance at p ≤ 0.01 based on Student’s t-tests between SPSL and Kongyu131.*

### Phenotypic Differences Among Plants With Different Cultivation Dates

**FIGURE 5:**
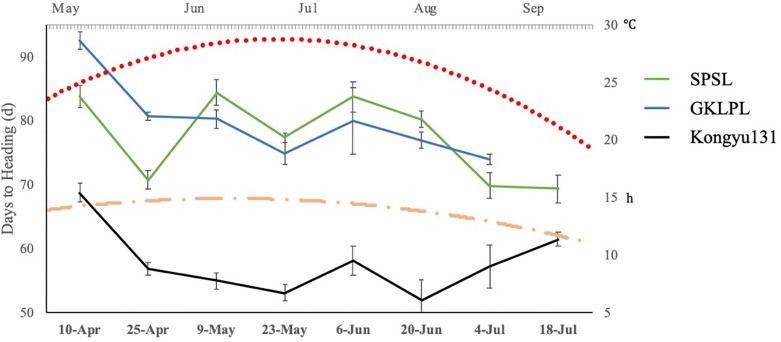
The trend of days to heading values with different sowing dates compared between the SPSL and its parents. The primary horizontal axis indicates the different sowing dates, and the primary vertical axis indicates the days to heading with different sowing dates. The blue line indicates the donor GKLPL (the last time points were missed); the green line indicates the SPSL; and the black line indicates the recurrent parent Kongyu131. Flat-ended arrows indicate standard deviation. The red dot line indicates daily mean temperature from May to September. The orange dot dash line indicates daily mean daytime from May to September.

Differences in DH may be affected by lengths of daytime and temperature. To examine this difference, we evaluated the SPSL with its parents as controls, in a normal paddy field in ZGQ with different sowing dates. The first seeding date was April 10th, with an interval of 14 days. The last seeding date was July 18th, with eight sessions in total. The daytime length in the experimental field peaked in June, with an average of 14.8 h of light per day. This duration gradually decreased to 12.4 h per day in September. Although the different sowing dates, all genotypes exhibited heading between June and September, indicating that all the varieties underwent heading under long-day conditions, as rice plants are affected by daytime length. The results showed that, regardless of seeding date, the DH value of the SPSL with the mRA7 module was greater than that of Kongyu131 (Figure 5). However, there was no linear correlation between daytime length and DH. According to the temperature monitoring data, the daily average temperature first increased but then decreased from May to September. The peak value was observed from July to early August. The DH of the SPSL exhibited no correlation with this temperature trend, but Kongyu131 exhibited an inverse correlation, i.e., the DH decreased at first but then increased. This finding suggests that the SPSL with the mRA7 module was substantially affected by both daytime length and temperature, but it was not temperature sensitive, unlike Kongyu131.

### The SPSL of mRA7 Adapted to a Change in Region From JNQ to ZGQ

To evaluate the phenotype of the SPSL in the natural field, the SPSL was planted in JNQ and other low-latitude areas, such as ZGQ and GZQ. The DH value of the SPSL was approximately 125 days in JNQ, which was similar to that of the local variety, exhibiting a significant difference compared to Kongyu131. When planted in ZGQ, the DH value of the SPSL was approximately 84 days, which was 15 days later than that of Kongyu131. Meanwhile, the PH and the LMP increased at both locations. Thus, the SPSL suitable for cultivation between the latitude of JNQ and ZGQ.

## Discussion

The aim of this work was to improve an elite rice variety for its regional adaptation with precise and efficient modularized improvement. In this study, we first conducted a QTL analysis with a BIL population constructed by using the GKMP cultivar as a donor, ensuring the absence of the regional adaptation locus in Kongyu131. Then we used a backcross population BC_3_F_2_ constructed by using GKLPL, a cultivar derived from GKMP as a donor to verify the QTL identified above and to attempt the introgression of such a locus in Kongyu131. Based on the position of the locus, we hypothesized that the *Ghd7* could be the candidate gene and we inferred that introduction of the *Ghd7* from GKLPL or GKMP to Kongyu131 activated its gene expression, thus delaying the HD of Kongyu131, and increasing the PH and yield-related traits, such as LMP and GNP. Then, the foreground and background were both selected from BC_4_F_1_ to BC_4_F_2,_ and we defined mRA7 the introgressed gene module. Finally, the SPSL was obtained with the precisely introduction of the mRA7 module in the BC_4_F_3_ generation (Figure 1). Evaluation of the module showed that the HD of SPSL was significantly delayed compared with that of Kongyu131, and the PH and LMP were increased (Table 5). All of these results indicated that introduction of the mRA7 module indeed improved the capacity of adaptation of the original Kongyu131. According to the phenotypic comparison between the SPSL and Kongyu131, we also verified that there were no non-desirable genes that perturbed the expected phenotype.

At the end, our research led to the development of a modular variety by using a single module, extending the cultivation of a main cultivar of Kongyu131 from its original area of cultivation (the third accumulative temperature belt) to low-latitude regions, such as JNQ and ZGQ. This method requires less time and resources than traditional breeding techniques. Notably, this method and technical route can be used as a reference for the transplantation of other main cultivars to non-original areas of cultivation.

### The mRA7 Module Could Correspond to the *Hd4*/*Ghd7* Gene

Based on genome resequencing and comparison, we could hypothesize that the Ghd7 is the candidate gene of the target QTL/gene module. Previous studies have shown that *Hd4*/*Ghd7* is a pleiotropic gene that controls the HD and yield-related traits such as PH, LMP and GNP. This gene encodes a CCT (CO, CO-LIKE and TIMING OF CAB1) domain protein. With overexpression, this gene can delay the HD under long-day conditions. Simultaneously, the LMP, PH, and GNP can increase significantly (Yano et al., 1997; Xue et al., 2008). Analogously, the SPSL showed a significant increase in PH, LMP, GNP, and NPB in the main panicle compared to Kongyu131. These phenotypic results may further indicate that the two donor parents may carry the functional allele correspond to the G*hd7* gene and thus that its expression could be the responsible of the change in phenotype of the SPSL in respect to the original Kongyu131. However, because the length of the module was approximately 800 kb, some other genes that affect the relevant phenotypes may be included, and further research is required to test this possibility.

### Yield Traits Might Not Be Affected by the Module

The delayed heading date in LQ had the consequences that the SPSL was exposed to low temperature during grain filling stage. That would be direct cause that the TGW of two donor cultivars and the SPSL was lower than Kongyu131, owing to incomplete grain filling under low temperature. In contrast, the SPSL heads at the right time in JNQ and ZGQ and TGW shows no difference between the SPSL and Kongyu131 in ZGQ. As we all known, yield traits could be complex and involve multi-gene actions. By comparing the yield traits of varieties cultivated in these environments, two possible explanations may be deduced. First, the introduction of this module did not affect the grain filling or the accumulation of nutrient substances. Second, the increase of DH led to higher level of photosynthetic product (also known as the source) in ZGQ. The GNP and LMP (also known as the library) increase simultaneous, indicating the presence of an equilibrium between the source and the library.

In both LQ and ZGQ, the ETP exhibited no differences between the SPSL and Kongyu131, indicating the mRA7 module did not affect the number of effective tillers. Therefore, this finding suggests that the extension of the adaptive region of Kongyu131 did not affect its production. The differences of both Kongyu131 and SPSL in these environments could be attributed to various environmental factors.

### Accurate Control of the Introduced Segment and Improving of the Genome

MAS-based breeding is widely used today based on the selection of linkage markers of target traits, to be combined with phenotypic selections in the field. However, the bred varieties from this technique are sometimes unstable, and the effective genes underlying may not be clear. After a few years’ cultivation, even a preeminent rice variety would be replaced for its defects. Unfortunately, breeders could do nothing to save it, but to develop new varieties with the same procedure. In our research, we successfully improved the genome of Kongyu131 by introducing and accurately controlling the size of the donor chromosome segment. First, we used a number of SNP markers to screen Kongyu131’s chromosomes and eliminate non-target fragments from the donor. Second, we used five SNP markers, namely, SNP1–SNP5, near and within the candidate gene. SNP3 was located in the gene sequence to ensure its introduction. The distance between SNP1 and SNP5 was approximately 1 Mb. With the two-step selection between SNP1 and SNP2 and between SNP4 and SNP5, the introduced segment was well controlled at less than 1 Mb. Traditional MAS-based breeding using a few scattered markers leads to uncertainty in the length of the introduced chromosome segment, often generating a large fragment insertion with the well-known effect of linkage drag.

Except for the precisely control of linkage drags and target genes, the availability of a gene module can allow the rough prediction of its effects upon introgression (Zeng et al., 2017; Kumar et al., 2018). This could be feasible also for high complex trait when main locus/genes responsible of are known or are mapped in the genomes. Therefore, this method can be used for directed and precise improvement of rice varieties.

### Extension of the SPSL and Its Prospects

To date, we have not identified any significant defects during field tests of our SPSL. However, extension of the SPSL to some of non-original cultivation areas requires consideration of the adaptability problems. In addition to the HD, several aspects, such as biotic and abiotic resistance, must be considered. If some defect would emerge after a few years’ cultivation, we could improve the trait by its related genes or QTL using a similar method. Then, two or more modules could be combined to upgrade the SPSL again. Due to the consistency of the genomic background of Kongyu131, we would simply need to hybridize the two SPSLs to obtain a multimodule improved variety. In addition, such kind of materials allow to compare homologous genes, gene interactions and the relationships of the genes with background genomes in a single genomic background. Therefore, our genetic system will be very important in both fundamental research and productive practice.

When attempting to develop a modular variety carrying more than one module, the most important factor to regulate would be the length of the single segment introduced in a variety. Recombination of different SPSLs may generate some unexpected phenotypes, owing to the interactions of genes that were introduced in the linkage drag accompanied with the target gene. The length of the linkage drag would be doubled by the aggregation of two modules. Hence, the control of the length of the introduced segment seems particularly important.

Overall, our research used high-density SNP markers covering the whole genome and introduced the regional adaptation-related mRA7 module from the donor GKLPL into Kongyu131. After elimination of the non-target chromosome fragments and precise control of the target module, an SPSL was obtained. The SPSL delayed the HD and significantly increased the length of the main panicle and the PH when cultivated in LQ, but also in JNQ and ZGQ, in respect to the original parent. We can estimate that this variety could be cultivated till more than 3 latitude units to the south.

## Author Contributions

SL conceived the experiments. RW carried out most of the experiments, performed the statistical analysis, and wrote the manuscript. XF, JN, and XZ participated in primer design. GJ and QY performed rice hybridization and field management. All authors read and approved the final manuscript.

## Conflict of Interest Statement

The authors declare that the research was conducted in the absence of any commercial or financial relationships that could be construed as a potential conflict of interest.

## Footnotes

1 www.ncbi.nlm.nih.gov/genbank

2 https://shigen.nig.ac.jp/rice/oryzabase/

3 https://blast.ncbi.nlm.nih.gov/Blast.cgi

## Abbreviations

DH: days to heading
ETP: effective tillers per plant
GN: grain number per plant
LQ: plots in Jiamusi, Heilongjiang province
GNP: grain number of main panicles
GZQ: plots in Guangzhou, Guangdong province
HD: heading date
HRM: high-resolution melting
JNQ: plots in Changchun, Jilin province
MAS: marker-assisted selection
NPB: number of primary branches
PH: plant height
QTL: quantitative trait loci
SNP: single-nucleotide polymorphism
SPSL: single point substitute line
ZGQ: plots in Beijing.
