# Supplementary figures and images for: Updating the Genome of the Elite Rice Variety Kongyu131 to Expand Its Ecological Adaptation Region
Source: Front Plant Sci. 2019 Mar 13;10:288. doi: 10.3389/fpls.2019.00288 (PMC6424915; doi:10.3389/fpls.2019.00288)

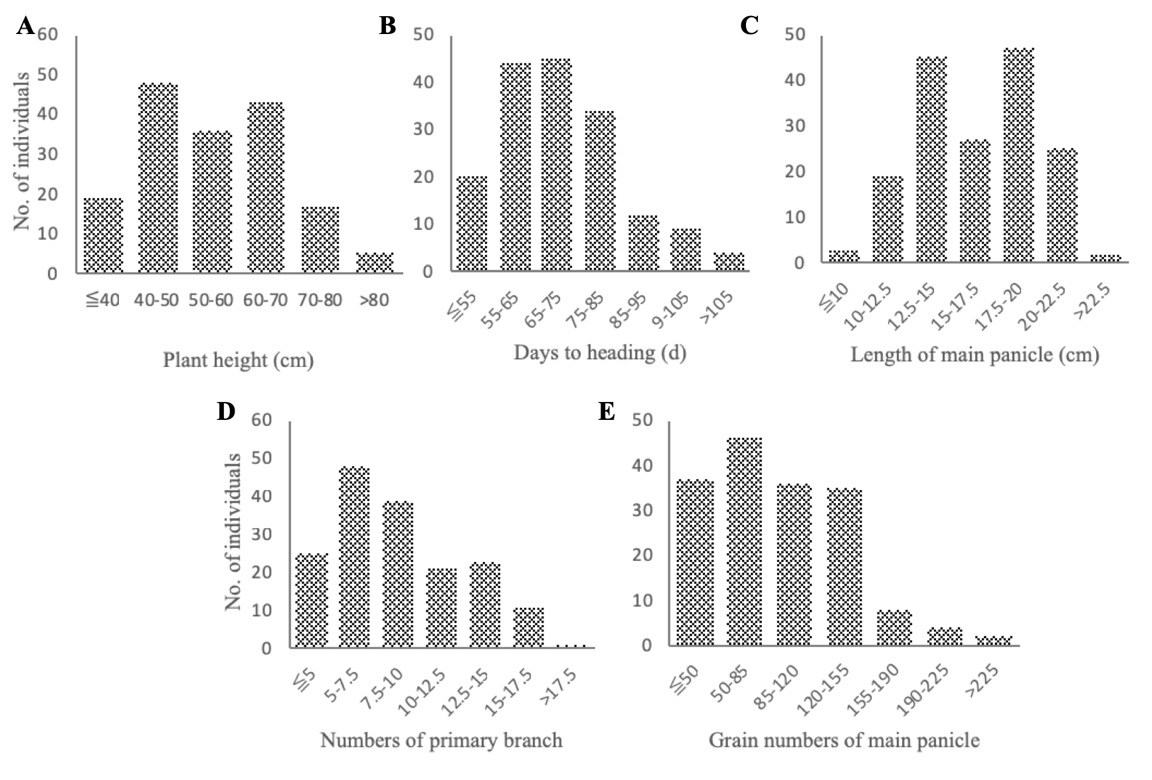

Supplement: Supplementary file 1 [file Image_1.JPEG]

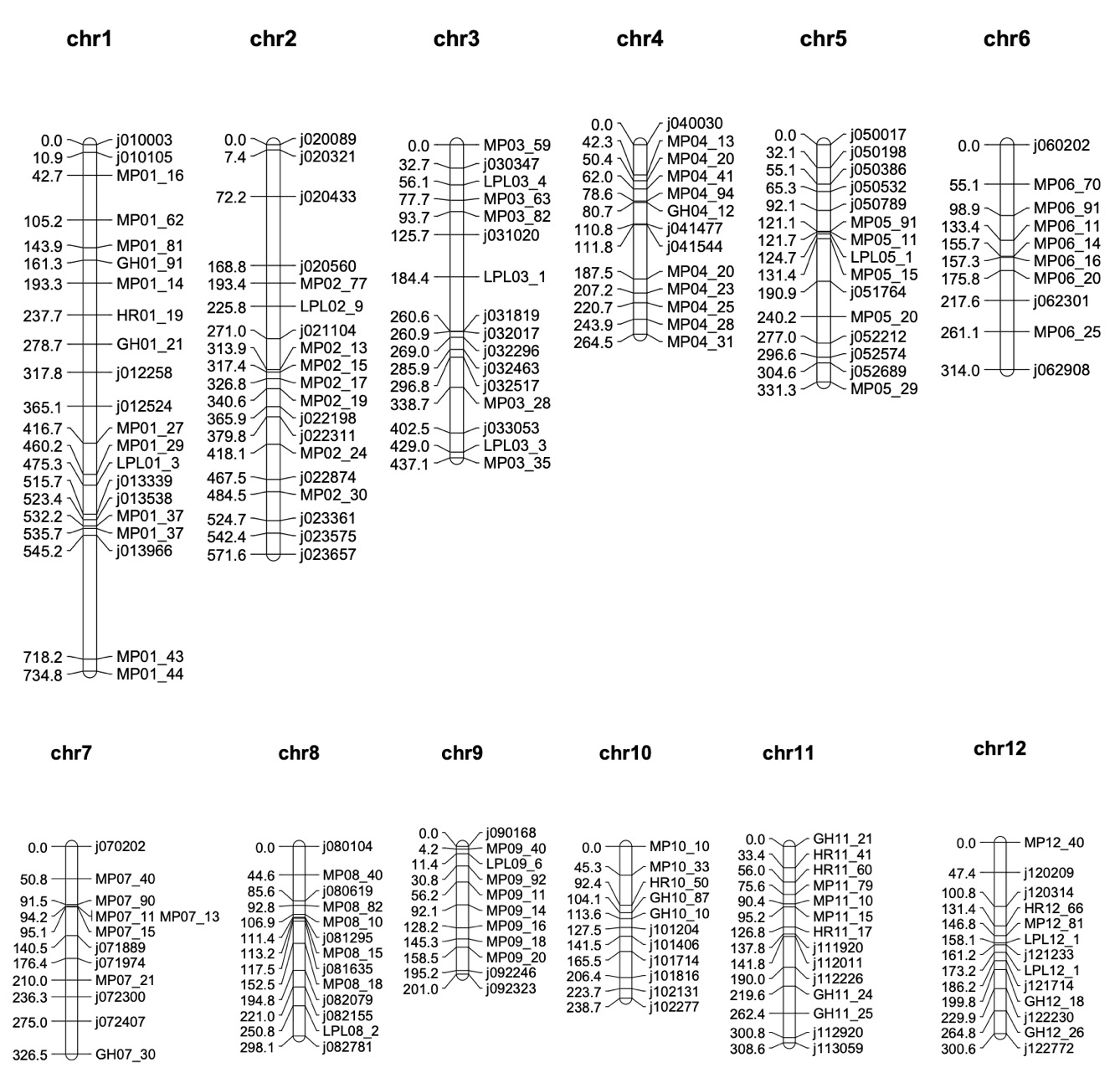

Supplement: Supplementary file 2 [file Image_2.JPEG]

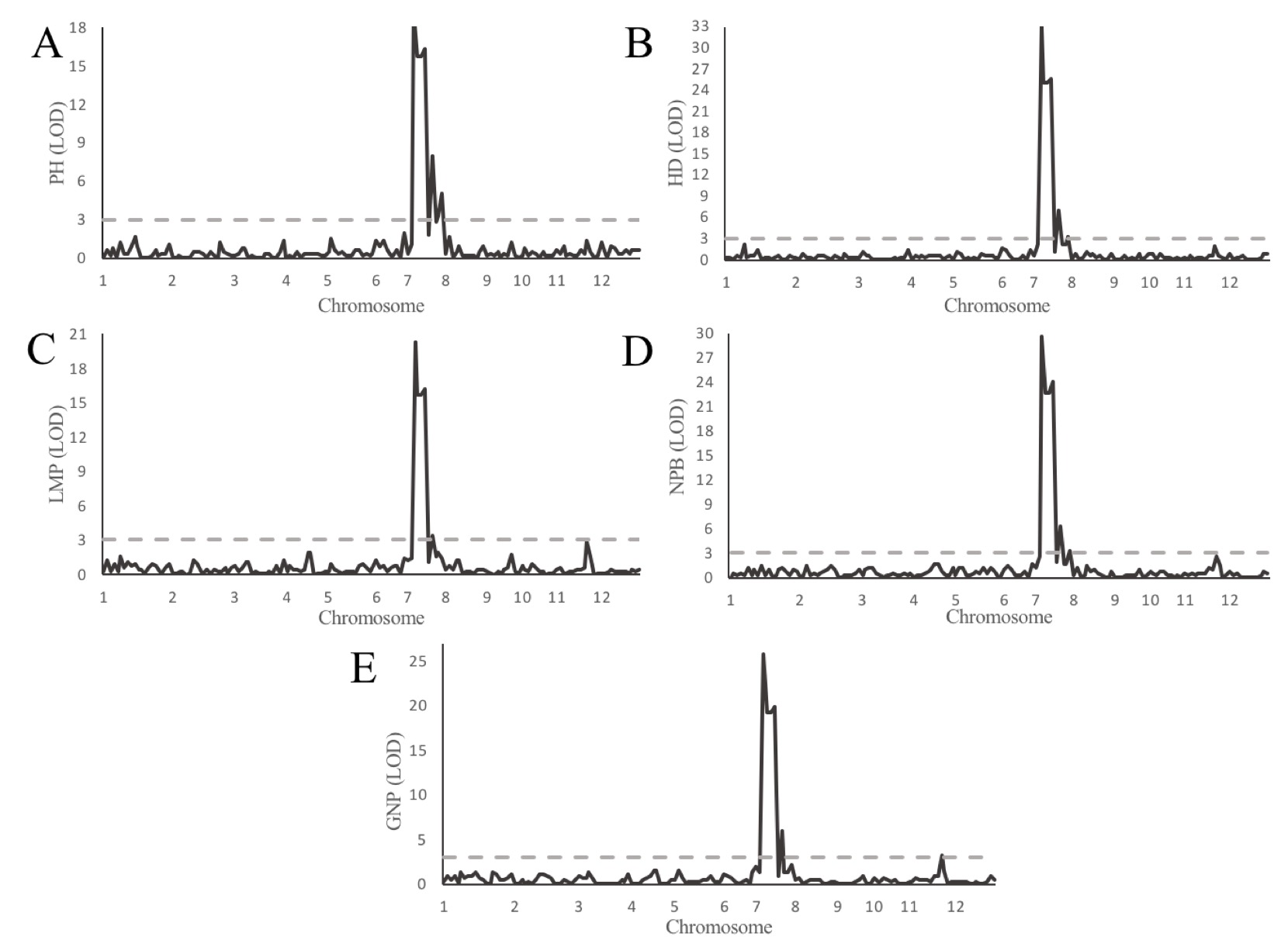

Supplement: Supplementary file 3 [file Image_3.JPEG]

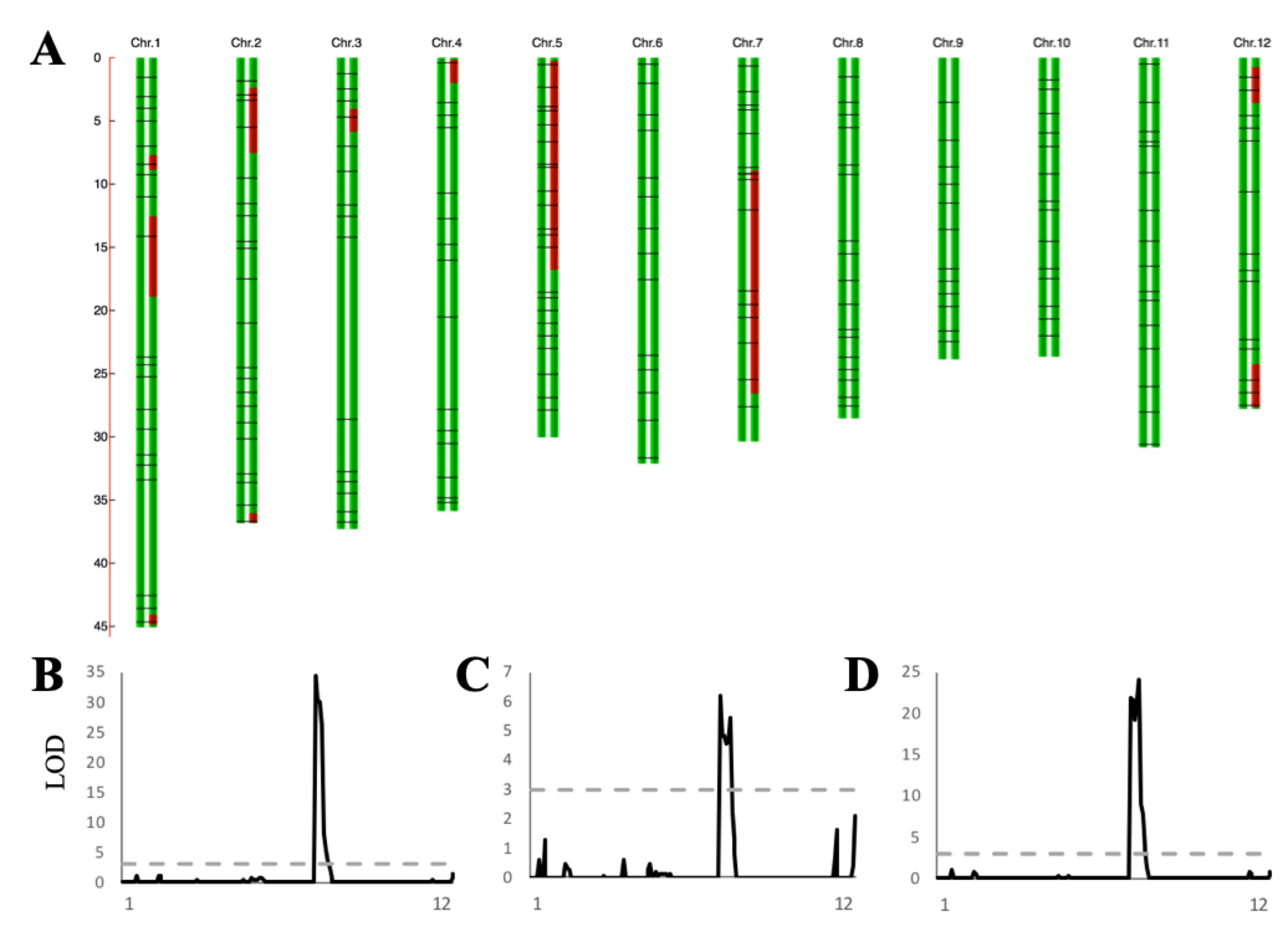

Supplement: Supplementary file 4 [file Image_4.JPEG]

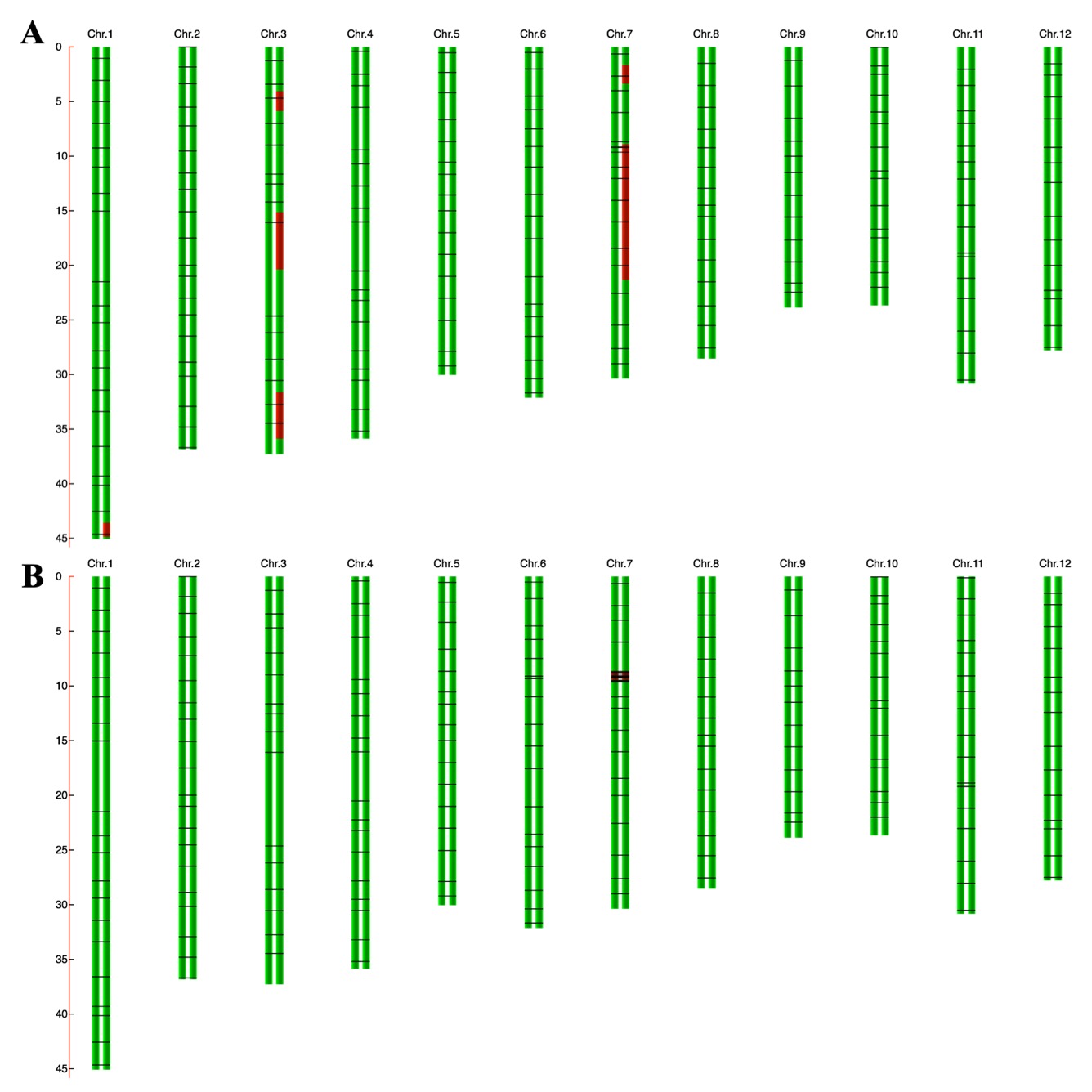

Supplement: Supplementary file 5 [file Image_5.JPEG]
