# Supplementary material for: Updating the Genome of the Elite Rice Variety Kongyu131 to Expand Its Ecological Adaptation Region
Source: Front Plant Sci. 2019 Mar 13;10:288. doi: 10.3389/fpls.2019.00288 (PMC6424915; doi:10.3389/fpls.2019.00288)
Supplement: Supplementary file 6 [file Table_1.DOCX]

**Fig. S1** Frequency distribution in the QTL population BC_1_F_6_-MP. The vertical axis indicates the number of plants; the horizontal axes indicates the **(A)** intervals of different plant heights, **(B)** intervals of different days to heading, (**C**) intervals of different length of main panicle, (**D**) intervals of different numbers of primary branch and (**E**) intervals of different grain numbers of main panicle.

**Fig. S2** Genetic map of BIL population BC_1_F_6_-MP

**Fig. S3** Results of QTL analysis of multiple traits in the BC_1_F_6_-MP population

LOD values derived from QTL analysis of the BC_1_F_6_ population based on the following five traits: plant height **(A)**, heading date **(B)**, length of main panicle **(C)**, number of primary branches **(D)** and grain number per panicle **(E)**.

**Fig. S4** Information regarding the BC_3_F_2_-LPL population. **(A)** Graphic depicting the genotype of BC_3_F_1_-LPL55F01. Green indicates the chromosome originating from the recurrent parent Kongyu131; red indicates the chromosome originating from the donor GKLPL; and the horizontal line indicates the positions of the SNP markers detected. **(B-D)** LOD score calculated from QTL analysis of the progenies of BC_3_F_1_-LPL55F01. The vertical axis indicates the LOD score, and the horizontal axis indicates the SNP markers distributed on 12 chromosomes with different traits: **(B)** days to heading, **(C)** length of main panicle, and **(D)** plant height.

**Fig. S5** Graphic depicting the genotype of the selected individual. **(A)** BC_4_F_1_-222E02 and **(B)** BC_4_F_3_-331E09. Green columns indicate the chromosome fragments from Kongyu131; red columns indicate the chromosome fragments from the donor GKLPL; and the horizontal lines indicate the positions of SNP markers used for genotyping. In the BC_4_F_1_-222E02 genome, only the end of the long arm in chromosome 1, chromosome 3 and the end of the short arm in chromosome 7 had some fragments from the donor.

**Table S1** Primer sequences for the SNP markers used for the mRA7 module

| Chromosome | SNP position | Name | Sequence |
| --- | --- | --- | --- |
| 7 | 8686544 | SNP1-Forward | CATAGAGGATTGCTATGGC |
|  |  | SNP1-Reverse | GAAGACGAGAGGTGGAAG |
| 7 | 9183364 | SNP2-Forward | GCACACCAACTAACAATGTC |
|  |  | SNP2-Reverse | GTAGTACTATTAGGAGTAAATGTC |
| 7 | 9186821 | SNP3-Forward | CGAGTGCGTGCCAGGGGATC |
|  |  | SNP3-Reverse | CGTTGCCGAAGAACTGGAAC |
| 7 | 9213272 | SNP4-Forward | CTTGACCTAATTTGATGAGTGG |
|  |  | SNP4-Reverse | AGGATCTCCTTGTGACTGTC |
| 7 | 9643247 | SNP5-Forward | ACGTCTCCGTCGTTGCTAAG |
|  |  | SNP5-Reverse | GCAACGACAGAAGCAACTGG |
| 7 | 8772090 | SNP1-1-Forward | CGACTCTTCGTTACTTTCTTGAAC |
|  |  | SNP1-1-Reverse | TACTCTTGCCATTGGAAGGATTAG |
| 7 | 8915167 | SNP1-2-Forward | CAGCAACGAGGAGGACTAAC |
|  |  | SNP1-2-Reverse | CTCACTACCGATGTTTCTCTCTC |
| 7 | 9085061 | SNP1-3-Forward | CAGAGCGTCTTGTTTAGAAGATGA |
|  |  | SNP1-4-Reverse | GAAGAAGATATCGTGGAGGACATA |
| 7 | 9293670 | SNP4-1-Forward | CTTCCATTGGACATGTAATCGTAC |
|  |  | SNP4-1-Reverse | AACCTAGCTTGTGCAACTACTT |
| 7 | 9479436 | SNP4-2-Forward | GTCTCTGACCGTCAATGGTG |
|  |  | SNP4-2-Reverse | TAGGAGAGAGCAGAGATGGGTTAG |
| 7 | 9567111 | SNP4-3-Forward | ATGCGCTCCTGTCGAGTA |
|  |  | SNP4-3-Reverse | GAACATTTGCTGTCACACCTG |

**Table S2** Correlation of different traits on QTL analysis population BC_3_F_2_-LPL

|  | LMP | PH |
| --- | --- | --- |
| PH | 0.5379 | 1 |
| DH | 0.5685 | 0.8284 |

DH, Days to heading; PH, plant height; LMP, length of main panicle.
